# Dataset for modeling the hydrological patterns and salinity fluctuations of a Mediterranean confined coastal lagoon system (La Pletera salt marsh area, NE Spain)

**DOI:** 10.1016/j.dib.2022.108593

**Published:** 2022-09-13

**Authors:** Warren Meredith, Xavier Casamitjana, Xavier D. Quintana, Anna Menció

**Affiliations:** a Grup de recerca en Geologia Aplicada i Ambiental (GAiA), Department of Environmental Sciences, University of Girona, 17003 Girona, Spain; b Department of Physics, University of Girona, 17003 Girona, Spain; c GRECO, Institute of Aquatic Ecology, University of Girona, 17003 Girona, Spain

**Keywords:** General lake model, Groundwater surface-water interaction, Constructed coastal lagoon, Water balance, Hydrology

## Abstract

This dataset is related to the research article entitled “Effects of morphology and sediment permeability on coastal lagoons’ hydrological patterns” (W. Meredith, X. Casamitjana, X. D. Quintana, A. Menció) [1], and was obtained in the La Pletera salt marshes in Catalunya between 2016 and 2019, to model the water balance and salinity fluctuations of 6 permanent lagoons using the General Lake Model (GLM). As no inflow and outflow data were available, water level and bathymetric data were used to calculate the net balance of inflows and outflows according to the observed daily volume fluctuations. Meteorological data were obtained from the L´Estartit Meteorological station north of the lagoons. Daily solar radiation was measured in Mas Badia (La Tallada, ∼10km from the La Pletera) in 2016 and 2017 and in situ with radiation sensors in 2018 and 2019. Together with the bathymetry and water levels of the lagoons, calculated inflows and calibrated salinity and temperature data are provided to further confined coastal lagoons system modeling where inflow and outflow data are not available. Meteorological data and observed lagoon salinity and temperature are provided for comparison. As this is one of the few datasets that have modeled coastal water bodies less than 3m in depth using the GLM, the data presented here can be useful in stress testing the General Lake Model to other coastal lagoon systems, as well as to other global aquatic ecosystems.

**Specifications Table**

**Table:** 

Subject:	Environmental Science
Specific subject area:	Hydrological modelling of coastal lagoons, Groundwater surface-water interaction, Lagoon morphometry, Restoration
Type of data:	Table Figure Excel File
How the data were acquired:	Procedures of collection are described in paragraph 3.1, 3.2 and 3.3 in Methods of Meredith et al., 2022 [1].
Data format:	Raw, Analyzed
Description of data collection:	Daily water levels from November 2014 to September 2017 were obtained from Schlumberger water level data loggers (accuracy ± 0.02 m) Water levels in 2018 and 2019 were obtained biweekly from depth gauge boards installed in the lagoons. Biweekly values for temperature and salinity were obtained from a CTD profiler (Sea & Sun Technology). Bathymetric data and water levels were used to calculate lagoon volume and corresponding water inflow quantities. Meteorological data was obtained from the L´Estartit meteorological station, 2 km north of the lagoons. Daily solar radiation was measured in Mas Badia (La Tallada, ∼10km from the La Pletera) in 2016 and 2017 and in situ with radiation sensors in 2018 and 2019.
Data source location:	• University of Girona • L'Estartit, Girona, Catalunya • Spain • 42.0513° N, 3.1905° E
Data accessibility:	Repository name: Figshare Direct URL to data: https://doi.org/10.6084/m9.figshare.20198804.v3
Related research article	W. Meredith, X. Casamitjana, X. D. Quintana, A. Menció, Effects of morphology and sediment permeability on coastal lagoons’ hydrological patterns, J. Hydrol. 612 (2022) 128259. https://doi.org/10.1016/j.jhydrol.2022.128259.

## Value of the Data

• This dataset provides information of the hydrology of a confined coastal lagoon system that is strongly influenced by irregular and unpredictable Mediterranean climatic events.
• This dataset is one of a few studies that have modeled coastal systems at a water depth lower than 3m.
• This dataset provides the basis to anyone who wants to use the General Lake Model in modeling confined coastal lagoons systems where inflow and outflow data are not available.
• The data can be useful in stress testing the General Lake Model to other coastal lagoon systems, as well as to global aquatic ecosystems.

## 1 Data Description

This dataset was used to model the water balance and salinity fluctuations of the La Pletera salt marshes in Catalunya. It forms the basis of core parameters required by the General Lake Model (GLM) to model water volumes, salinity and temperature profiles of the six lagoons labeled; BPI, FRA, G02, L01, L04, M03. As this is a confined coastal system, it was not possible to measure inflows or outflows and they had to be calculated manually from the fluctuating volume levels of each lagoon. Table 1 is the bathymetry data that calculate the volumes of the lagoons at any single depth using a polynomial fit described in [1]. Table 2 are the GLM physical parameters used in modeling the La Pletera salt marshes. Because of the small volume and shallow depth of the lagoons, the minimum values for volume and thickness of the Lagrangian layers are established one order of magnitude smaller than the typical values. The physical parameters C_Κ_, η_∗_, C_Τ_, C_S_ and C_HYP_ are related to the individual mixing process efficiencies and don´t require calibration; their values are based on observations, experiments in the laboratory and theoretical deliberation [2], and are set to the usual values.

**Table 1:** Bathymetry of the lagoons of the La Pletera salt marshes

	Surface Area (m^2^)					
Height above sea level (m)	BPI	FRA	G02	L01	L04	M03
1.5		17290	2991			
1.25			2700			
1	5387	16733	2500	8657		8007.08
0.9				7732		
0.8				7059.33		5000
0.7	1383.72	14478	2000	3492.85	9878	4000
0.6						3041.1
0.5	435.83	13605	1550		9060.63	2810.47
0.4						2589.57
0.3				2684.69	7732	2375.15
0.25	288.58	12154	1500			
0.2				2322.05	6399.4	2154.33
0.1				1827.29	4428.9	1906.65
0	213.31	2530.12	1491	1512.18	2950.08	1630.66
-0.1				1068.26		
-0.2	138.12			0	1606.16	500
-0.3					883.58	0
-0.4					585.51	
-0.5	60.12	1516.16	500		217.5	
-0.6	0				0	
-0.75			300			
-1		1056.78	250			
-1.5		259.39	0			
-1.6		0

**Table 2:** The values of the GLM physical parameters used in modelling the La Pletera salt marshes.

Mixing and thermodynamic parameters		
C_K_	Mixing efficiency-convective overturn	0.2
η	Mixing efficiency-wind stirring vs convection	1.23
C_S_	Mixing efficiency-shear production	0.23
C_T_	Mixing efficiency-kinetic requirement	0.51
C_HYP_	Mixing efficiency-hypolimnetic mixing	0.5

*Model structure*		

Maximum Lagrangian layers		200 m^3^
Minimum layer volume		0.025 m^3^
Minimum layer thickness		0.005 m
Maximum layer thickness		0.05 m

In the excel files “InputDataGLM.xlsx” there are 9 excel sheets:

• The matrices of the first 6 sheets are the input data of inflow, temperature and salinity of each of the lagoons required by the GLM. These have been calibrated to fit the observed volumes, temperatures and salinity. It is important to note these files need to be converted .csv to run in the GLM.
• The seventh sheet is the observed water levels of the lagoons in centimeters above sea level (note that the bottom of the lagoons are below sea level).
• The last two sheets are the biweekly observed salinity and temperature values between 2016 and 2019 of the six lagoons.

**Fig. 1:**
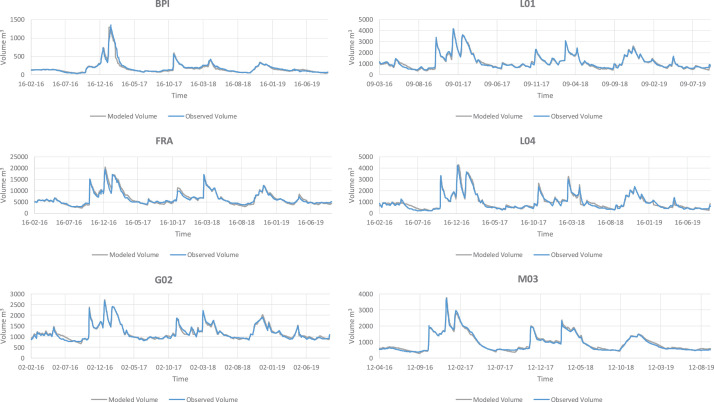
Modeled vs observed values for volume of all the lagoons in the La Pletera salt marsh.

**Fig. 2:**
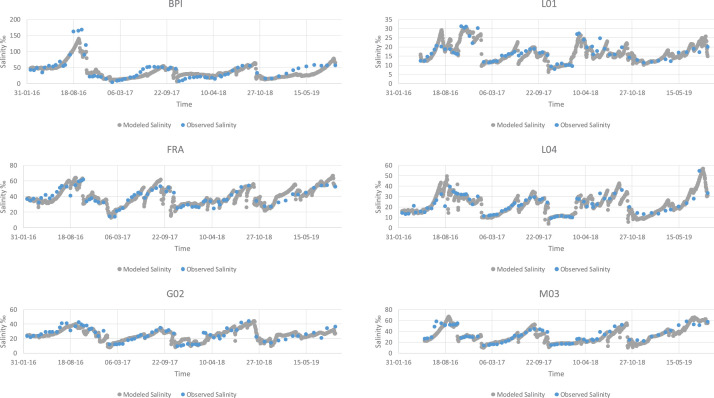
Modeled vs observed values for salinity of all the lagoons. Salinity is measured in parts per thousand.

**Fig. 3:**
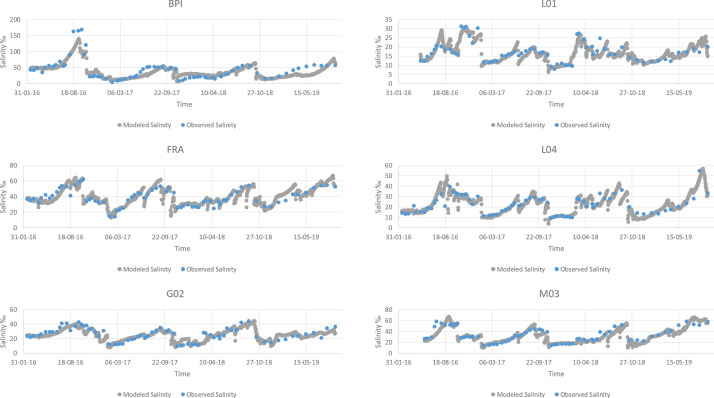
Modeled vs observed values for temperature of all the lagoons.

**Fig. 4:**
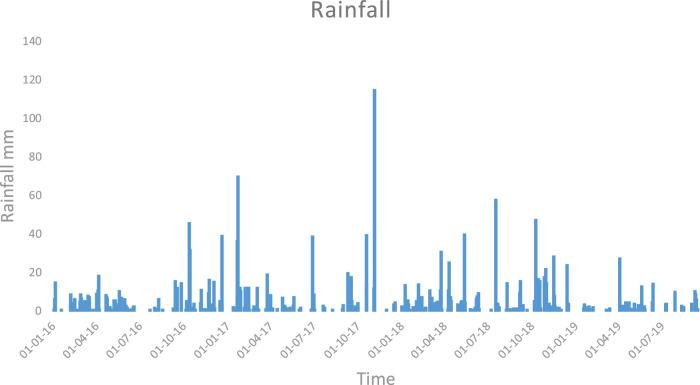
Rainfall in the La Pletera salt marsh between 2016 and 2019.

The excel file “MeteoData.csv” is the meteorological data of the La Pletera salt marshes during the study period and is required by the GLM. Data includes short wave radiation, cloud cover, air temperature, relative humidity, wind speed and rain. The last column is snowfall and is not applicable in this coastal system. This file is applicable to all the lagoons listed here. The resulting modeled vs. observed values for volume, salinity and temperature are depicted in Figs. 1–3, respectively. Rainfall between 2016 and 2019 is shown in Fig. 4 and can be used to compare with volume fluctuations of the lagoons. The lagoons also receive surface inflow from seawater inputs during winter cyclonic storms, as well as subterranean water flows during the summer period, when there is usually no rainfall. These inflows are modeled separately from rainfall.

## 2 Experimental Design, Materials and Methods

The measurements for water levels were taken daily by Schlumberger water level data loggers (accuracy ± 0.02 m) in 2016 and 2017 and biweekly from depth gauge boards installed in the lagoons in 2018 and 2019. Biweekly data for temperature and salinity were measured by a sonde (CTD) profiler (Sea & Sun Technology). The L´Estartit meteorological station, 2 km north of the lagoons, provided daily-average measurements of relative humidity, precipitation, and maximum and minimum temperatures. Daily solar radiation was measured in Mas Badia (La Tallada, ∼10km from the La Pletera) in 2016 and 2017 and in situ with radiation sensors in 2018 and 2019. Inflow and outflow measurements were estimated from the water levels of the lagoons. As there were no inflow and outflow measurements, the volumes were estimated using a polynomial fit [1], and the net daily inflows and outflows were calculated according to the volume fluctuations observed. The modeled inflows and outflows were then set from the calculated net daily inflow and outflow calculations. Rain and evaporation fluxes are modeled separately from the inflows and outflows. As a result, the inflows and outflows were adjusted until the modeled and real volume, temperature, and salinity values showed the smallest possible differences through an iterative process.

When modeling the water balance, salinity and temperature fluctuations, the General Lake Model incorporates inflows/outflows, mixing, and surface heating and cooling, and calculates vertical profiles of temperature, salinity, and density with a flexible Lagrangian layer structure [3,4]. These layers contract or expand according to inflows, outflows, surface mass fluxes and mixing. From the total daily inflow and outflow data and daily-average meteorological data, the surface momentum, sensible heat, and latent heat fluxes are computed from the following bulk aerodynamic formulae for the stress τ (Νm^−2^), the sensible heat transfer H (Wm^−2^), and the evaporative heat transfer E (Wm^−2^)

(1) $$\tau =\rho_{A}C_{D}U^{2}$$

(2) $$H=-\rho_{A}C_{P}\;C_{H}U\left(T_{A}-T_{S}\right)$$

(3) $$E=-\rho_{A}\;L_{V}C_{W}U\left(q_{A}-q_{S}\right)$$

where ρ_Α_= air density; U= wind speed; T= air temperature; q= specific humidity (all daily averaged); and subscripts A for air and S for water surface values. C_H_, C_W_ and C_D_ are bulk aerodynamic transfer coefficients, and are determined by the height where the data were taken. C_P_ is the specific heat of water at constant pressure and L_V_ is the latent heat of evaporation of water. The water mass evaporation can be calculated from E/Lv of the lagoons and is in kg m^−2^ s^−1^.

The short wave radiant flux that is distributed through the water column is calculated using Beer's law formulation

(4) $$Q\left(z\right)=Q_{o}e^{-\eta z}$$

where Q_o_= measured radiation at the surface; Q(z)= the intensity at depth z, and η= the light attenuation coefficient. The constant light attenuation factor was set to 1.7 m^−1^, which is a typical value for eutrophic waters [5]

An integral turbulent kinetic energy model forms the basis for surface layer dynamics [2] and is divided into four discrete processes: wind stirring, convective overturn, interfacial shear production, and Kelvin-Helmholtz billowing. The model calculates the energy available through each of these processes and is a function of the nature of the stratification and the strength of the forcing. This is then compared with the potential energy required to combine the mixed layer with the layer immediately below. The layers are mixed by averaging their properties if sufficient energy is available. This is repeated until not enough energy remains within the present time step to continue the deepening process. This residual energy is then added to the available energy in the next time step. The parameterization for the available turbulent kinetic energy (KE_A_) is:

(6) $$KE_{A}=\frac{C_{K}}{2}\left(w_{*}^{3}+\eta_{*}^{3}u_{*}^{3}\right)\Delta t+\frac{C_{S}}{2}\left(u_{1}^{2}+\frac{u_{1}^{2}}{6}\frac{d\delta }{dh}+\frac{u_{1}\delta }{3}\frac{du_{1}}{dh}\right)\delta h$$

and for the required potential energy (PE_R_) is:

(7) $$PE_{R}=\frac{C_{T}}{2}\left[{\left(w_{*}^{3}+\eta_{*}^{3}u_{*}^{3}\right)}^{\frac{2}{3}}+\frac{\Delta \rho gh}{\rho_{o}}+\frac{g\delta^{2}}{24\rho_{o}}\frac{d\left(\Delta \rho \right)}{dh}+\;\frac{g\Delta \rho \delta }{12\rho_{o}}\frac{d\delta }{dh}\right]\delta h$$

where u_*_ and w_*_= velocity scales for wind shear and penetrative convection, respectively; u_1_ = shear velocity at the surface; Δρ= density jump between the surface layer (with depth h) and the layer immediately below it (with depth dh); ρ_ο_ is a reference density; δ is the Kelvin-Helmholtz billow thickness scale; Δt = the time step; and g= the acceleration due to gravity.

The turbulent diffusivity coefficient, D_z_, is used to model hypolimnetic mixing. This depends directly on the dissipation of the turbulent kinetic energy and inversely on the stratification. The formula is based on Weinstock (1981), and is given by the expression

(8) $$D_{z}=\frac{C_{HYP}\varepsilon }{N^{2}+ut_{*}^{2}k_{o}^{2}}$$

where ko is the wave number of the largest eddies; ut* is the turbulent velocity scale; ε is dissipation; and C_HYP_ is a constant related to the mixing efficiency of the turbulence.

## Ethics Statements

The authors declare there are no ethical issues with the data presented or methods used.

## CRediT authorship contribution statement

**Warren Meredith:** Conceptualization, Formal analysis, Visualization, Writing – original draft. **Xavier Casamitjana:** Methodology, Conceptualization, Writing – review & editing. **Xavier D. Quintana:** Conceptualization, Writing – review & editing. **Anna Menció:** Conceptualization, Supervision, Writing – review & editing.

## Declaration of Competing Interest

The authors declare that they have no known competing financial interests or personal relationships that could have appeared to influence the work reported in this paper.

## Data Availability

Dataset for modelling the hydrological patterns and salinity fluctuations of a Mediterranean confined coastal lagoon system. (Original Data) (Figshare).
